# A large-scale study of a poultry trading network in Bangladesh: implications for control and surveillance of avian influenza viruses

**DOI:** 10.1186/s12917-018-1331-5

**Published:** 2018-01-12

**Authors:** N. Moyen, G. Ahmed, S. Gupta, T. Tenzin, R. Khan, T. Khan, N. Debnath, M. Yamage, D.U. Pfeiffer, G. Fournie

**Affiliations:** 1 Department of Pathobiology and Population Sciences, Royal Veterinary College, University of London, Hatfield, Hertfordshire AL9 7TA UK; 2 School of Veterinary Science, The University of Queensland, Gatton, 4343 Qld Australia; 3 Emergency Centre for Transboundary Animal Diseases, Food and Agriculture Organisation of the United Nations, Dhaka, Bangladesh; 4 National Centre for Animal Health, Thimphu, Bhutan; 5 College of Veterinary Medicine and Life Sciences, City University of Hong Kong, Tat Chee Avenue, Kowloon, Hong Kong

**Keywords:** Poultry network, Bangladesh, Surveillance, Avian influenza

## Abstract

**Background:**

Since its first report in 2007, avian influenza (AI) has been endemic in Bangladesh. While live poultry marketing is widespread throughout the country and known to influence AI dissemination and persistence, trading patterns have not been described. The aim of this study is to assess poultry trading practices and features of the poultry trading networks which could promote AI spread, and their potential implications for disease control and surveillance.

Data on poultry trading practices was collected from 849 poultry traders during a cross-sectional survey in 138 live bird markets (LBMs) across 17 different districts of Bangladesh. The quantity and origins of traded poultry were assessed for each poultry type in surveyed LBMs. The network of contacts between farms and LBMs resulting from commercial movements of live poultry was constructed to assess its connectivity and to identify the key premises influencing it.

**Results:**

Poultry trading practices varied according to the size of the LBMs and to the type of poultry traded. Industrial broiler chickens, the most commonly traded poultry, were generally sold in LBMs close to their production areas, whereas ducks and backyard chickens were moved over longer distances, and their transport involved several intermediates. The poultry trading network composed of 445 nodes (73.2% were LBMs) was highly connected and disassortative. However, the removal of only 5.6% of the nodes (25 LBMs with the highest betweenness scores), reduced the network’s connectedness, and the maximum size of output and input domains by more than 50%.

**Conclusions:**

Poultry types need to be discriminated in order to understand the way in which poultry trading networks are shaped, and the level of risk of disease spread that these networks may promote. Knowledge of the network structure could be used to target control and surveillance interventions to a small number of LBMs.

**Electronic supplementary material:**

The online version of this article (10.1186/s12917-018-1331-5) contains supplementary material, which is available to authorized users.

## Background

Bangladesh has a human population of more than 145 million resulting in a density of 1072 people per km^2^, and an estimated national poultry population of 304 million resulting in a poultry density of about 2400 poultry per km^2^ [1, 2]. About 75% of the Bangladeshis live in rural areas and depend heavily on poultry, both as a source of proteins and of income: 1.8 million people are involved in poultry farming alone [2], and, in 2013, poultry meat represented half of the country’s meat production [3]. As a result, a zoonotic infectious disease affecting the Bangladeshi poultry production system could have a large impact on the country’s food security, economy and public health.

The first highly pathogenic avian influenza subtype H5N1 (HPAI H5N1) outbreak in Bangladesh was reported in 2007 [4, 5]. This strain is now considered to be endemic in the country [6, 7]. Live bird trading and marketing have been shown to play a major role in the maintenance of avian influenza viruses (AIVs) within a number of poultry production systems [8, 9]. Yet, in Bangladesh, live bird trading is ubiquitous, as more than 90% of poultry are marketed through live bird markets (LBMs) [10]. Previous studies conducted in other settings have identified an association between LBM characteristics (such as the number of poultry traded, trading frequency, and the number of poultry traded with other LBMs) and the risk of dissemination of AIVs [11–13]. In addition, the networks shaped by commercial poultry movements, within which LBMs generally act as hubs, have also been shown to support the dissemination and maintenance of avian viruses such as Avian Influenza (AI) and Newcastle disease [13–15].

In Bangladesh, migratory birds have been associated with the introduction and spread of HPAI H5N1 [16], but poultry trade and trade-related activities such as the exchange of egg trays between farms, or the introduction of contaminated vehicles into farms have also been identified as potential sources of AIV infection for commercial and backyard poultry flocks [5]. The HPAI H5N1 outbreaks that occurred between 2007 and 2009 in Bangladesh were spatially clustered along the country’s main highways and principal poultry trading routes, which also supports the role of poultry trading activities in the spread of viruses through transport of infected poultry or contaminated material and vehicles [16–19]. Nevertheless, so far, national poultry trading networks have not been described, nor has their potential role in virus spread been assessed. In order to address this knowledge gap, a cross-sectional survey was conducted throughout the country to assess practices of live poultry traders, and to characterise the structure of the networks resulting from the trade of live poultry.

In Bangladesh, husbandry systems and the geographic location of poultry farms are strongly associated with the type of poultry reared. The four main poultry types traded in the country are: Industrial white-feathered broiler chickens (such as Hybro-PN, Hubbard classic, Ross, Cobb 500 [10]), sonali poultry (crossbreed between a Fayoumi female and a Red Island Red male [20]), deshi (local chickens raised in backyards [21]) and ducks. The first two poultry types are raised in commercial farms, and almost 70% of the commercial flock is located in the two most densely populated divisions of Bangladesh. Whereas deshis and ducks are raised in traditional scavenging systems, and more than a third of the backyard flock is located in one rural division [10, 22–24]. We therefore hypothesised that live poultry trading practices and networks may vary according to the types of poultry. While live poultry trading networks have been described in multiple settings [14, 25–28], they have not been characterised according to poultry types. Yet, identifying the types of poultry traded through these networks may provide insights to understand how these networks may generate disease risk for poultry and human populations.

## Methods

### Data collection

**Fig. 1:**
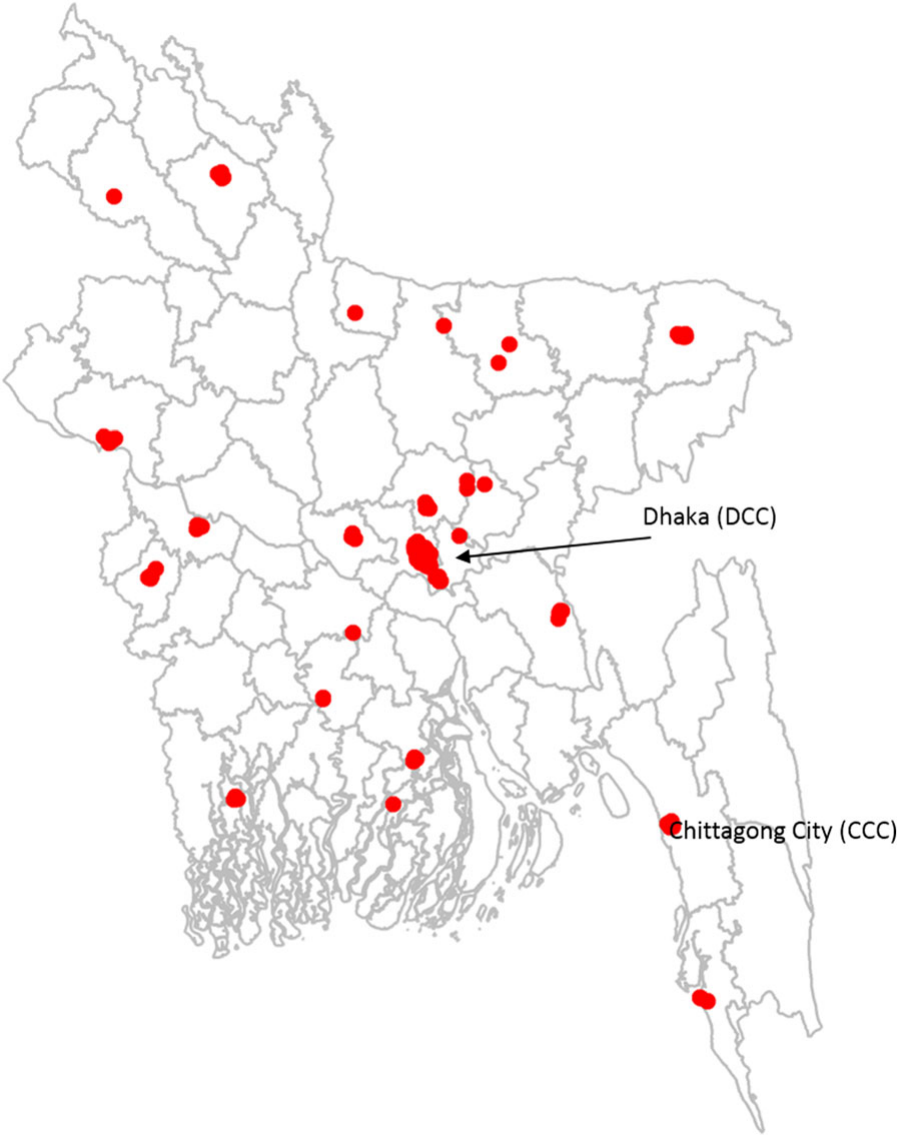
Location of the 138 surveyed LBMs included in the study. One week data, collected from 849 traders in 2014

A trader was defined as a person whose main activity is to buy poultry from other poultry traders or farmers and to sell it either to other poultry traders or consumers. In order to select the LBMs in which the poultry traders would be interviewed a multi-stage purposive sampling process was followed. Bangladesh is divided into 64 districts (second administrative division) and 490 upazilas (sub-districts, third administrative division). A cross-sectional survey was carried in 138 LBMs across 17 upazilas purposively selected, located in 17 different districts of Bangladesh in September 2014. These 17 upazilas included the 2 main urban centres of the country, Dhaka City Corporation (DCC) and Chittagong City Corporation (CCC) which represent about 15% of the total human population of the country. The 15 other upazilas were selected based on their proximity to previous reported AI outbreaks, their high poultry density and their proximity to international borders. Within each upazila, LBMs identified with the highest quantity of traded poultry by local experts were recruited. Of the recruited LBMs, 50 (36%) were located in Dhaka district, all in DCC. In the 16 other selected districts, 1 to 16 LBMs were visited (Fig. 1). Once the LBMs selected, the traders were selected either randomly or purposively depending on if they operated in the LBM permanently or not. In LBMs where less than 5 traders operated permanently, all traders were interviewed. In LBMs with 6 to 10 permanent traders, 50% of them were randomly selected, and in LBMs where more than 10 permanent traders were present, 30% of them were randomly selected. In addition, as many traders (often referred to as middlemen) supplying permanent LBM traders as possible were also interviewed. As a result, a total of 849 poultry traders were interviewed. Verbal consent was obtained from the interviewees prior to the interviews. Standardised questionnaires were administered in Bengali by trained interviewers and Global Positioning System (GPS) coordinates of the surveyed LBMs were recorded. Informants were asked about their trading practices in the week preceding the interview: number of poultry sold to other poultry traders or consumers, number of poultry bought, types and locations from which poultry were sourced. Although traders remembered the names and towns of the LBMs they bought from, this was not the case for the farms, for which only the upazilas were given. Some traders could not identify the origin of their poultry: they reported having bought poultry from a trader who had bought from another trader. As these transactions represented only 1.2% of the total number of poultry traded through this network, and they did not affect the overall structure of the network, they were not considered in the analysis. All the data was collected according to poultry types. However, for clarity, and given the small proportion of traded poultry that they represented (5.5%), spent hens, geese, pigeons and quail were grouped into an “other poultry type” category. The 4 main poultry types considered here are: industrial white-feathered broiler chicken, sonali poultry, deshi and ducks.

### Statistical analyses and social network analysis

Data was entered in Microsoft Excel 2013® (Microsoft Corp., Redmond, WA, USA), and analyses were conducted in R 3.2.5 [29].

Network terminology and metrics are defined in Table 1.

**Table 1:** Definitions of network terminology and metrics used in this study

Poultry sources	1) “Farm upazila”: An upazila (third administrative division) in which farms supplied a trader in a surveyed LBM. Exact names and locations of farms were unknown, they were therefore grouped into so-called “farm upazilas”. 2) A LBM (each LBM was an independent location).
Network node	A surveyed LBMs, a “farm upazila” or a non-surveyed LBM.
Network arc	Link between 2 nodes, weighted with the number of poultry traded between the considered sources and destinations.
Giant weak Component (GWC)	The largest subset of nodes in which all the nodes were connected, regardless of the direction of the arcs.
Giant Strong Component (GSC)	The largest subset of nodes in which all the nodes were connected, accounting for the direction of the arcs.
Connectedness of the network	The proportion of nodes included in the GWC [30].
Normalised betweenness-centrality	The proportion of shortest paths (i.e. geodesic distances) on which a given node lies.
Geodesic distances	Shortest path between two nodes, with the distance being calculated as the sum of the inverse of the arc strengths [64].
Input domain	Proportion of the nodes that can reach a node following network arcs.
Output domain	Proportion of the nodes that can be reached by a node following network arcs.

The total number of poultry traded per LBM could not be estimated because the total number of poultry traders operating permanently or not at the surveyed LBMs was unknown. We therefore used the average weekly number of poultry traded per trader in a surveyed LBM (i.e. the total number of poultry traded by all the traders interviewed in a LBM in the past week divided by the number of traders interviewed in that LBM).

The number of poultry traded per trader, the number and types of poultry sources and the number of LBMs that an interviewed trader supplied were summarised using median and inter-quartile ranges (IQR). The surveyed LBMs were then compared with respect to the practices of their traders: proportion of poultry supplied according to the type of source, number of different poultry sources per LBM, number of upazilas of origin of the poultry traded at the LBM, and distribution of transaction distances. A transaction distance was the Euclidean distance between a poultry source and a surveyed LBM. It was weighted by the number of poultry traded between these two locations. It was calculated using the GPS coordinates of the surveyed LBMs and the centroid of the upazilas in which farms and non-surveyed LBMs supplying surveyed LBMs were located, as their GPS coordinates were not available. Thus, non-surveyed LBMs and farms located in the same upazila had the same coordinates. The relative importance of the effect of within and between LBM variance on the number of poultry traded per trader was assessed using a mixed effects model with surveyed LBMs as random effects.

Weighted and directed networks were built for each poultry type. A node was defined as either the group of interviewed traders of the surveyed LBMs or a “farm upazila”, and each network arc was weighted with the number of poultry traded between the considered sources and destinations. In order to assess the network connectivity, as well as the potential lower and upper bounds of potential epidemic sizes [30], the sizes of the giant weak and strong components (GWC and GSC) were calculated for each type of network described. For LBMs, the average weekly number of poultry traded by a trader was calculated as a measure of node centrality. Normalised betweenness-centrality was calculated for each node, accounting for arc strength. The directed network’s assortativity coefficient was calculated to assess the network’s resilience to targeted removal of LBM nodes. Indeed, disassortative networks, in which high degree nodes are preferentially connected to low degree nodes, are less resilient to node removal then assortative networks, in which nodes are preferentially connected to other nodes with similar degree [31, 32]. While the output domain may be interpreted as the potential of a given node to spread a pathogen, and, therefore, be an indicator of the node suitability as a target for control measures, the input domain may indicate nodes which should be targeted by surveillance programs. These two metrics were calculated for each node. Finally, in order to assess the impact of targeted control measures on the spread of AIVs, the effect of targeted node removal on network connectedness, the size of maximum output and input domains was assessed. Nodes to be removed were selected according to their betweenness score, the size of their input and output domains. Removal of LBM nodes can be interpreted as the implementation of cleaning, disinfection and rest day programmes in the given LBMs [14, 15, 33]. Such measures would result in the local elimination of viruses, so that arcs leading to and starting from such a node would then not be infectious any more.

The following R packages were used for the above mentioned analyses: “lme4” [34], “sp” [35, 36], “maptools” [37], “shapefiles” [38], “sna” [39], and “igraph” [40].

## Results

### Traders and LBM characteristics

Half of the interviewed traders reported having traded at least 1250 (IQR: 600–2945) poultry during the previous week. The total number of poultry traded weekly by interviewed traders in surveyed LBMs is presented in an Additional file 1. As the proportion of traders interviewed per surveyed LBM was not recorded, the total number of poultry traded in these LBMs could not be estimated. We therefore present the average number of poultry traded per trader and the proportion of trade represented by each poultry type. Broiler chickens were the main poultry type traded, they represented 42% of all the poultry sold by interviewed traders. In contrast, deshi and sonali chickens accounted for 19% and 33%, respectively, and ducks for only 0.5% of the interviewees’ poultry trade. About 90% of interviewed traders sold at least 2 poultry types. Sixty-five percent of traders traded broilers, almost half traded deshi and/or sonali, and only 4.5% traded ducks (Table 2).

**Table 2:** Number and types of poultry traded by surveyed traders and at LBMs in the week preceding the interviews

		Broiler	Sonali	Deshi	Ducks	Others	All
Trader level	Proportion of traders selling each poultry type (%).	64.9% (*n* = 551)	47.9% (*n* = 407)	46.5% (*n* = 395)	4.5% (*n* = 38)	26.9% (*n* = 228)	100% (*n* = 849)
	No. of poultry traded per week per trader interviewed^a^(median and IQR^b^).	1000 (420–2170)	650 (250–1575)	450 (200–1000)	50 (40–275)	400 (150–800)	1250 (600–2945)
	Proportion of a trader’s sales represented by each poultry type^a^(median % and IQR).	77.5% (47.4–100)	40% (21–59.3)	38.5% (19.6–66.7)	5.5% (2.6–18.9)	24.6% (12.6–40)	NA
LBM level	Proportion of LBMs in which a type of poultry is sold (%).	94.9% (*n* = 130)	76.8% (*n* = 106)	71% (*n* = 98)	14.5% (*n* = 20)	48.6% (*n* = 67)	100% (*n* = 138)
	Proportion of poultry of a given type sold in each LBM (median % and IQR).	52.9% (31.8–78.8)	14.7% (1.6–33.6)	9.1% (0–23.6)	0% (0–0)	0% (0–12.3)	NA
	Proportion of traders trading each type of poultry in a given LBM (median % and IQR).	85.2% (50–100)	40% (17.6–80)	35.4% (0–75)	0 (0–0)	0 (0–50)	NA
	Average no. of poultry sold per week and per trader in a given LBM^a^ (median and IQR^b^).	900 (337–2029)	296 (140–1106)	183 (82–696)	29 (14–75)	223 (71–328)	1800 (766–3303)

One-week data, collected in Bangladesh in 2014, from 849 traders in 138 LBMs

In this table “LBM” refers to the group of interviewed traders from the surveyed LBM and cannot be generalised to the entire LBM

^a^Including only the traders/LBMs which sold these types of poultry

^b^Inter-quantile range

Broiler chickens were sold in almost all LBMs, deshi and sonalis in 70% and 75% of LBMs, respectively, and ducks in 14.5% of LBMs. In half of the surveyed LBMs, at least 85.2% of the interviewed traders traded broilers, at least 35.4% traded deshis and at least 40% sonalis (Table 2).

Using a mixed effects model, with LBMs as random effects, 65% of the variance in the number of poultry traded per trader was estimated to be due to within-LBM variance. However, when only considering the 25% largest LBMs (*n* = 34), 98% of this variance was explained by within-LBM variance.

The distributions of the number of poultry sources (i.e. “farm upazilas” or LBMs) per interviewed trader and per LBM were right-skewed. Interviewed traders reported having up to 13 different poultry sources (median: 2, IQR: 1–3) in the past week, resulting in some surveyed LBMs having at least 24 different sources, as not all sources were identified given the sampling strategy (median: 4, IQR: 2–7) (Table 3). The number of different sources was positively correlated with the number of traders interviewed per LBM (Spearman’s correlation coefficient: 0.64, *p*-value < 10^−15^). In 20 % (*n* = 25) of the surveyed LBMs the interviewed traders were exclusively supplied by farm upazilas. These LBMs were distributed throughout the country; all of the interviewed traders in these LBMs sold broilers, 44% of them sold sonalis and 36% sold deshis, none of them sold ducks. In contrast, in 39.3% (*n* = 48) of the surveyed LBMs interviewed traders were supplied exclusively by other LBMs. Forty of those LBMs were in DCC, in 88% of them the interviewed traders sold broilers, in 73% of them they sold sonalis, in 71% of them they sold deshis, and in 6% of them they sold ducks.

**Table 3:** Proportion and types of poultry sources, according to poultry type for surveyed traders and LBMs

	Broiler	Sonali	Deshi	Ducks	Others	All
No. of poultry sources/trader (median and IQR^a^).	2 (1–3)	1 (1–2)	1 (1–2)	1 (1–1)	1 (1–1)	2 (1–3)
No. of poultry sources/LBM (%).	3 (2–4)	3 (1–5)	3 (1–5)	1 (1–2)	2 (2–4)	4 (2–7)
Proportion of surveyed LBMs supplied exclusively by other LBMs (%).	34.6% (n = 48)	46.7% (*n* = 64)	61.4% (*n* = 85)	71.4% (*n* = 99)	37.3% (*n* = 51)	39.3% (*n* = 54)
Proportion of surveyed LBMs supplied exclusively by farm upazilas (%).	32.8% (*n* = 45)	24.3% (n = 34)	14.9% (*n* = 21)	23.8% (n = 33)	28.4% (n = 39)	20.5% (*n* = 28)
Proportion of surveyed LBMs supplied by other LBMs and farm upazilas (%).	32.8% (n = 45)	29% (n = 40)	23.8% (n = 33)	4.8% (*n* = 7)	34.3% (*n* = 47)	40.2% (n = 55)
Proportion of poultry supplied to a LBM by another LBM (% and IQR).	48% (0–100)	78% (11.8–100)	100% (52.8–100)	100% (12.5–100)	61.5% (0–100)	57.6% (13.5–100)
Proportion of poultry supplied to a LBM by a farm upazila (% and IQR).	51% (0–100)	19.6% (0–76.4)	0% (0–29.7)	0% (0–37.5)	38.5% (0–100)	40.7% (0–79.4)
Number of different upazilas of origin of the poultry sold at surveyed LBMs (median and IQR).	2 (1–3.5)	2 (1–3)	2 (1–3)	1 (1–2)	2 (1–3)	3 (1.3–5)

One-week data, collected in Bangladesh in 2014, from 849 traders in 138 LBMs

Only the traders or the LBMs trading the poultry type considered were included in the calculations. In this table “LBM” refers to the group of interviewed traders from the surveyed LBM and cannot be generalised to the entire LBM

^a^Inter-quantile range

In at least 70% of the surveyed LBMs, more than half of ducks and deshis supplied to the interviewed traders were supplied by other LBMs. In contrast, broilers were supplied to the interviewed traders of the surveyed LBMs equally by other LBMs and farms (Table 3).

**Fig. 2:**
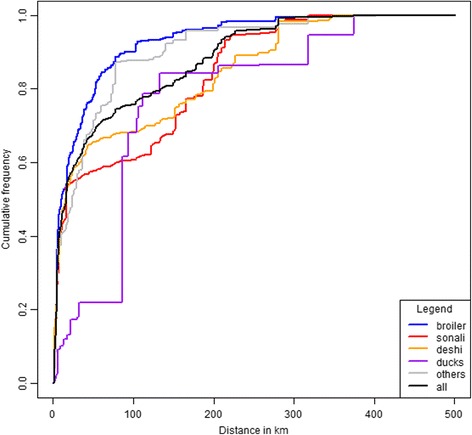
Cumulative distances (distances are weighted) between poultry sources and surveyed LBMs according to poultry type. One-week data, collected in Bangladesh in 2014, from 849 traders in 138 LBMs

Overall, half of the poultry supplied to the interviewed traders were sourced less than 15 km away from the surveyed LBMs, but this distance could reach up to 420 km (Fig. 2). While most poultry, regardless of their type, were sourced in the vicinity of the LBMs in which they were sold, the tail of the distribution of the distances over which they were transported varied according to the poultry type considered. Broiler chickens were sourced the closest to the LBMs (25% of all broilers were transported over more than 40 km, and 3% over more than 200 km), deshis and ducks the furthest (25% of all the deshis and ducks were transported over more than 105 km and 16% over more than 200 km).

In only 41% of the surveyed LBMs did interviewed traders report supplying other LBMs, and in 24% (*n* = 33) of those LBMs, interviewed traders only supplied a unique LBM. In 4 surveyed LBMs, interviewed traders sold to more than 8 other LBMs, 3 of these LBMs were in DCC and one was in the N-E of Bangladesh (Sylhet district).

### Poultry trading networks

In the networks described here, nodes were either farm-upazilas or the group of interviewed traders of each surveyed LBM, later referred to as “LBM”.

**Fig. 3:**
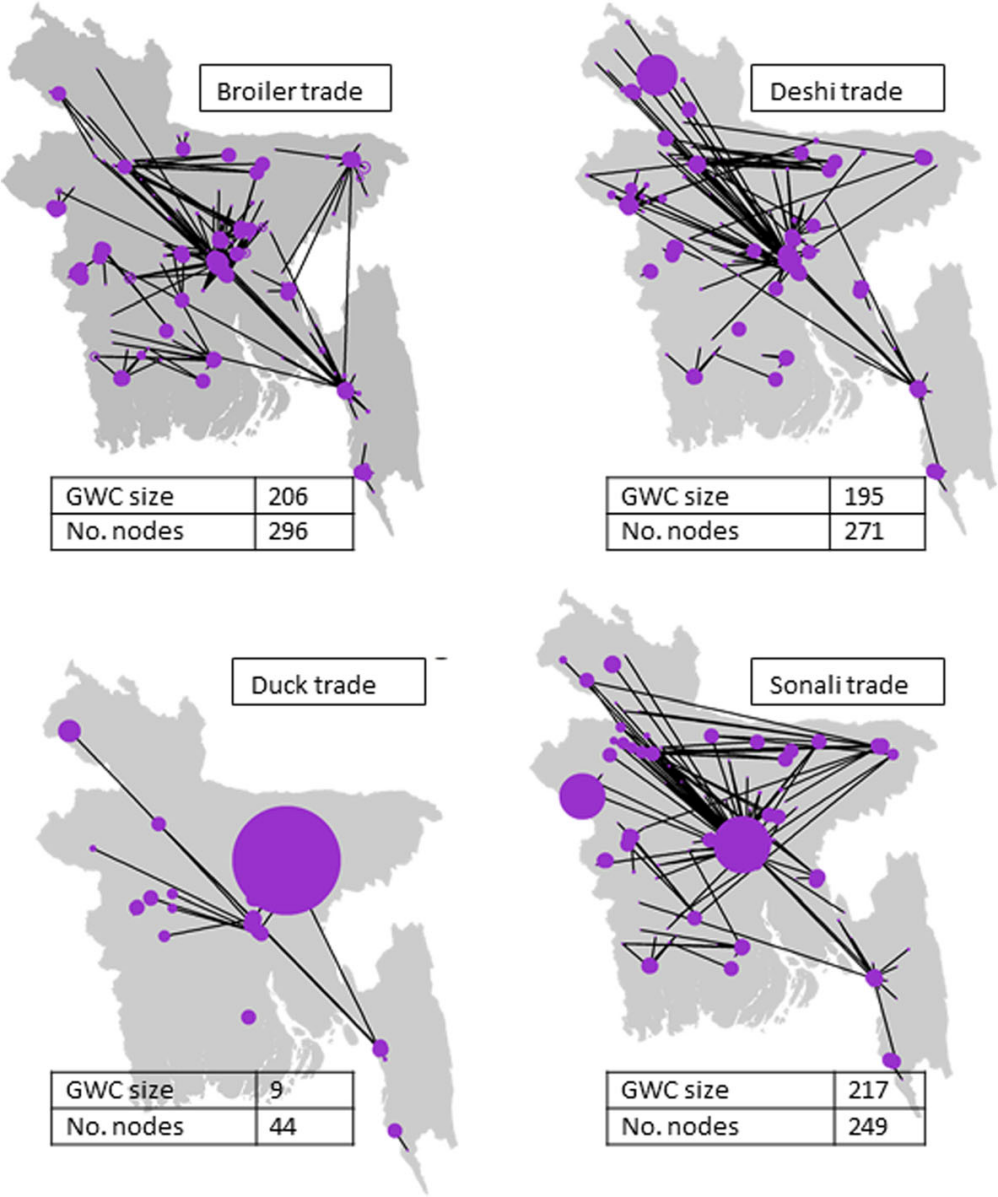
Poultry trading networks according to the type of poultry traded. The size of each dot is proportional to the number of poultry it supplies to the network. The direction of the arcs is not shown. One-week data, collected in Bangladesh in 2014, from 849 traders in 138 LBMs

As a result of the heterogeneity in trading practices, the trading networks differed according to poultry types. In the broiler trading network, 69.5% (206/296) of nodes were encompassed within the GWC. Sonali and deshi trading networks had similar levels of connectedness, 87% and 72% respectively. The duck trading network was the smallest and the least connected of the 4 trading networks represented in Fig. 3, with only 20.5% nodes encompassed within the GWC. The largest sources of sonalis for the interviewed traders were located in the centre and far north-west of Bangladesh, and deshis were mainly sourced from the west of the country. In this study, interviewed traders did not source broilers from a specific geographical location.

**Fig. 4:**
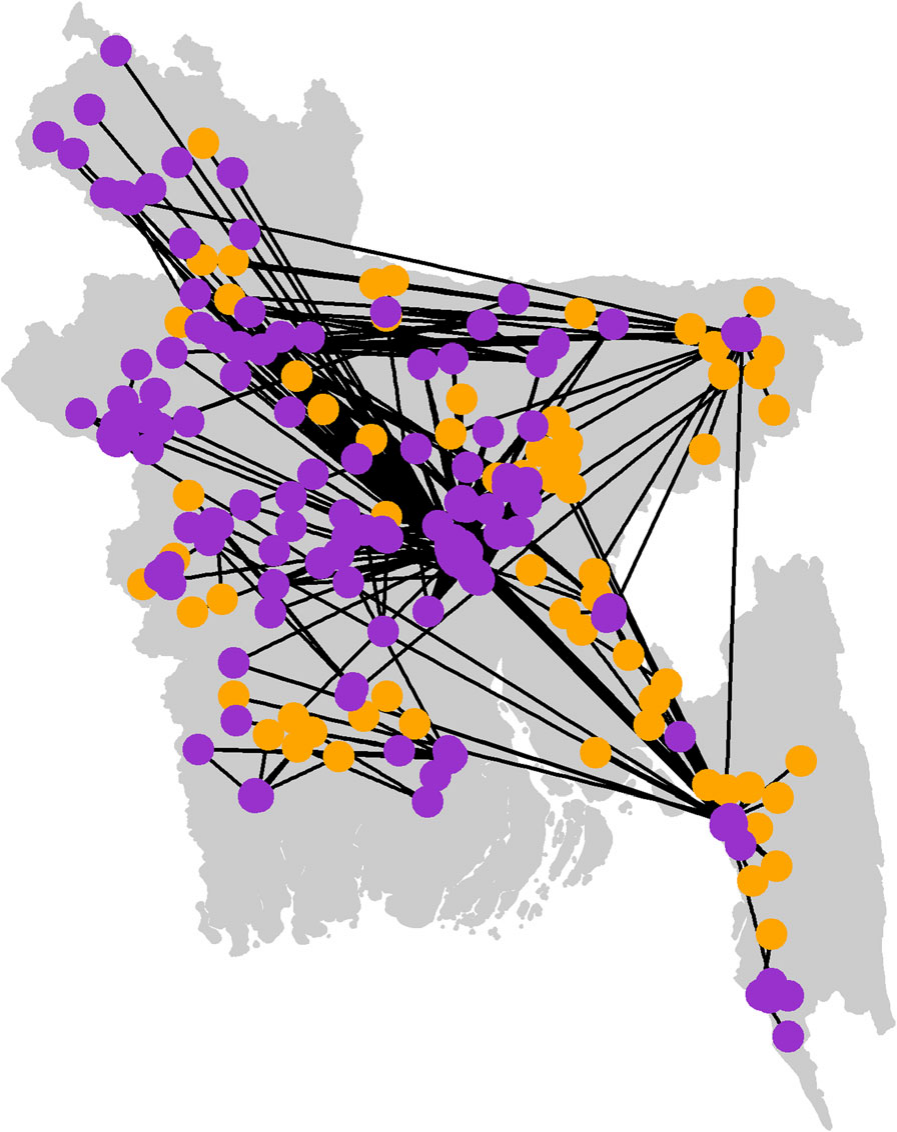
Poultry trading network. Nodes are LBMs (*purple*), or farm upazilas (*orange*). When the GPS coordinates of the nodes were not available (for all the nodes that are not the surveyed LBMs) the GPS coordinates of the centroid of the upazila were used. The direction of the arcs is not shown. One-week data, collected in Bangladesh in 2014, from 849 traders in 138 LBMs

When considering all poultry types, the network size and connectivity increased: it was composed of 445 nodes (Fig. 4), of which 73.2% were LBMs, and it was highly connected, with the GWC encompassing 97% (*n* = 433) of those nodes. The 12 remaining nodes were LBMs and farms in the South-East of the country (Cox’s Bazar district), all encompassed within another component. This region was however connected to the GWC through other LBMs. The GSC only grouped 2 nodes, reflecting the strong directionality of poultry commercial movements through the network.

The poultry trading network described here was disassortative (−0.27), therefore unlikely to be resilient to targeted node removal. Input and output domains were right-skewed with median values of 0 (IQR: 0–0.011) and 0.004 (IQR: 0–0.012) respectively; 159 nodes had an input domain greater than 0, 324 nodes had an output domain greater than 0. Of all the LBMs included in the network, 8.9% had an input domain greater than 5%, and 2.2% had an input domain greater than 10%. Only 4% of the LBMs or the farm upazilas had an output domain greater than 5%, and 2.5% of them had an output domain greater than 10%.

Thirty-eight surveyed LBMs (28% of the total number of surveyed LBMs, and 8.6% of all the nodes of the network) lay between 2 other nodes. Their betweenness was positively correlated with the size of the output domain (Pearson’s correlation coefficient: 0.90, *p*-value < 10^−15^). The LBM with the greatest betweenness score was a large wholesale LBM in DCC that supplied 6.1% of all the poultry traded through the network. The LBM with the 2nd greatest betweenness score was outside DCC and was supplied by the farm upazila with the largest output domain.

**Fig. 5:**
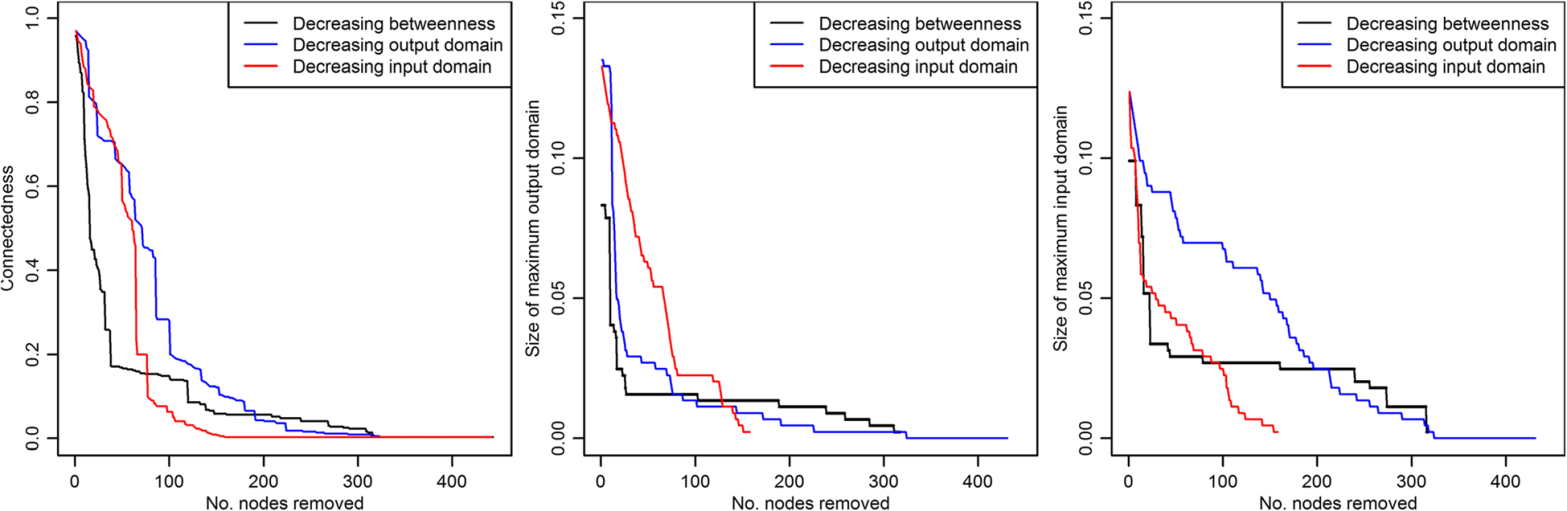
Comparison of the impact of LBM removal on network metrics (maximum output and input domains and connectedness). Nodes were removed from the network one after the other, in decreasing order of their betweenness, output or input domains. One-week data, collected in Bangladesh in 2014, from 849 traders in 138 LBMs

Comparing the impacts of node removal on the maximum output and input domains and the connectedness revealed that the removal of the nodes with the greatest betweenness scores would have the greatest impact on the network’s connectedness, and maximum output and input domains (Fig. 5). Removing 25 LBMs (5.6% of the entire network) decreased the maximum size of the input domain by 66%, the maximum size of the output domain by 73% and the connectedness by 58%. These nodes were LBMs located throughout the country (including in DCC and CCC). They all sold broilers, sonalis and deshis and 3 of them also sold ducks.

## Discussion

To the best of our knowledge, this study represents the first assessment of poultry trading practices and networks on a nationwide scale in Bangladesh. Trading patterns varied according to the type of poultry being traded. Broilers were the main type of poultry sold by interviewed traders and they were sourced from both farms and other LBMs located in the vicinity of the surveyed LBMs. In contrast, sonalis, deshis and ducks were mainly sourced from other LBMs and were mainly bought further away. Most deshis came from the North-west of Bangladesh, ducks from the North of the country, and sonalis from the central and North-Western districts. The overall poultry trading network was highly connected and disassortative. Removing a small fraction of nodes with the highest betweenness scores substantially reduced the networks’ connectedness and maximum sizes of output and input domains.

This study had some limitations. Firstly, we used the centroid of the upazilas as a proxy for the exact coordinates of farm upazilas and non-surveyed LBMs. As the exact GPS coordinates were unavailable for farms and non-surveyed LBMs, using upazila centroids as coordinates provided an estimate of the range of transaction distances according to poultry types traded. However, since upazilas can be as big as 300 to 400 km^2^; the resulting measurement error varies between observations in this dataset. Secondly, the sampling strategy impacted the estimation of the network’s metrics and their interpretation. As the proportion of traders interviewed in each LBM was unknown, some LBM-level metrics, such as the total number of poultry traded per LBM (weighted indegree), and the total number of arcs sent to and from a LBM (unweighted indegree and outdegree), could not be calculated. In addition, LBMs cited by interviewed traders as poultry sources were not surveyed, leading to the construction of an incomplete network, the total size of which was unknown. The proportion of connections identified in large LBMs was likely to be lower than in small LBMs as the proportion of interviewed traders was generally lower in large than in small LBMs. Therefore, the centrality measures of the largest LBMs might have been underestimated. Likewise, the impact of the removal of these nodes on network connectedness could have been underestimated. Nevertheless, the number of surveyed LBMs was large compared to other similar studies [26, 41, 42], and although network metrics might have been underestimated, the network properties highlighted here are likely to reflect relevant features of the real, complete network as well as poultry type-specific trade patterns.

Within-LBM variance in the number of poultry sold by traders was higher in the largest LBMs. This could be due to greater heterogeneity in traders’ practices in large LBMs compared to small LBMs. Homogeneity of trading practices seemed indeed more likely in small LBMs, where only a limited number of middlemen come to sell small numbers of poultry. This pattern should be taken into account in the design of future surveys. A large proportion of traders operating in the largest LBMs would need to be surveyed in order to identify most poultry trading routes, and to appropriately assess the diversity in trading practices. On the other hand, homogeneity in the practices of traders operating in small LBMs would mean that characteristics of non-surveyed small LBMs can be extrapolated based on a small survey sample.

This survey was conducted over the period of a month, it was therefore impossible to identify temporal variation in trading practices or network structure. However, in multiple settings, the number of traded poultry was reported to increase during seasonal and religious festivals, such as Chinese New Year celebrations and Ramadan [25, 26, 42, 43]. A study in China [42] also reported that during Chinese New Year celebrations, not only did the number of poultry traded increase, but so did the number of arcs and distances over which poultry was traded. Such trading patterns are likely to occur in Bangladesh as well, during religious festivals such as Ramadan and Eid-ul-Adha for example. They may particularly impact the trade of deshis, which are considered a delicacy. While increases in trading activities would be expected to promote AIV spread, as observed in Viet Nam [43], alteration of network structures may promote the mixing of poultry from a variety of geographical origins and farming systems and the reassortment of viral strains which otherwise might have remained isolated. Other factors that are likely to alter the structure of the poultry trading network include temporal variation in the market price of finished poultry or production inputs, e.g. day-old-chicks, and the occurrence of natural disasters, e.g. floods. Moreover, since duck production is linked to rice harvesting, duck trade is also likely to be seasonal [10]. Future investigations should aim at quantifying temporal variation in trading patterns for each poultry type in order to explore their impact on AIV epidemiology.

The distribution of the distances travelled by poultry between their sources and the LBMs surveyed in this study, were similar to those reported in other studies conducted in South-East Asia [11, 26, 44] and New Zealand [45]. Only in China were the maximum distances travelled by poultry greater than those described in our study [42]. In the first two HPAI H5N1 epidemic waves that occurred in Bangladesh in 2007–2008, clustering was seen within distances of 250-300 km [16]. In addition, genetically identical viruses caused outbreaks over an area of more than 200 km in 2007, indicating long-distance transmission events within Bangladesh [46]. Long-distance viral spread was also reported in 2010–11 [47], with movement of infected poultry or contaminated materiel being suggested as possible transmission routes. Transaction distances estimated in this study support the aforementioned results, and the possible role played by poultry trade in long distance spread of AIVs. In our study, cross-border trade was not reported. Nevertheless, such activities cannot be ruled out, as illegal importation of poultry across the porous Indian border has been mentioned in a previous study [48]. Cross-border trade would be worth investigating further in order to address the role of poultry trade in regional spread of AIVs.

The high level of contamination of Bangladeshi LBMs with a variety of AIV strains [49–53] and the association between LBM density and the risk of HPAI H5N1 outbreaks in Bangladeshi farms [18] suggests that AIV surveillance and control programmes implemented in LBMs could be effective for reducing disease risk for the production sector, as well as for humans. Furthermore, the position of nodes, either live animal markets or farms, in networks of potentially infectious contacts has been associated with their roles in the spread of pathogens [14, 54]. Consequently, the knowledge of the network structure can be used to help identifying the most suitable targets for control and surveillance programmes. Poultry trading networks described in multiple settings were all heterogeneous, with a small number of LBMs having a major influence on the potential of viruses to spread through these networks [8, 33, 55–57]. Targeting surveillance and control programmes at this specific set of LBMs is likely to be the most cost-effective and realistic control option, especially in resource-poor settings such as Bangladesh. In this study, the removal of 25 LBMs (5.6% of all the nodes of the network) substantially reduced the network’s connectedness, and the maximum size of input and output domains. We would therefore expect that the implementation of control measures, such as daily cleaning, disinfection and regular rest days [33, 58] in these LBMs to reduce the potential of pathogens to spread through the network. Indeed, reducing the maximum size of the output domain would decrease the potential of any contaminated LBM to spread viruses through poultry trade movements, and a reduced connectedness would reduce the maximum possible size of an epidemic. LBMs with the largest number of poultry sources and input domain received poultry from the most geographically diverse locations throughout Bangladesh. As a limited number of LBMs exhibited these features, targeting surveillance activities to these LBMs could allow the monitoring of the diversity of AIV strains circulating in the country. In addition, if control measures were implemented throughout the network, a reduction in viral diversity in LBMs could be an indicator of the effectiveness of control measures. However, even if a small number of LBMs is to be targeted, the effective implementation of such control strategies may prove challenging. Although poor hygiene in Bangladeshi LBMs was associated with a higher likelihood to detect AIVs in one study [52], studies have shown that only a limited number of biosecurity measures have been implemented in LBMs so far [49, 59]. Furthermore, one study revealed that those limited changes had not been sufficient to reduce the level of AIV contamination in LBMs with better biosecurity measures compared to LBMs with less or no biosecurity measures in place [49]. These results suggest a suboptimal implementation of biosecurity measures. In order to ensure that control interventions are effective in the future, further studies should aim to assess their feasibility, their economic impact and the compliance of LBM traders. These interventions may need to be complemented by a reinforcement of biosecurity measures at farm-level and during poultry transportation from farms to LBMs, with the aim to reduce the viral load introduced into LBMs. Indeed, poultry management and infrastructure of small commercial chicken farms did not meet basic biosecurity requirements. In particular, vehicles were regularly allowed on farm premises without prior disinfection [60].

In Bangladesh, HPAI H5N1 was more frequently detected in ducks than in chickens [48, 53], and in deshi than in broiler chickens [48]. In a recent study, Bangladeshi LBM stalls selling ducks alongside other poultry types were more than twice as likely to test positive for AIVs as stalls that didn’t sell any ducks [52]. While ducks are known to play an important role in HPAI H5N1 spread and maintenance, in particular due to their ability to remain asymptomatic [61–63], the structure of the Bangladeshi poultry trading network may further foster their impact on the epidemiology of the disease. Most deshis and ducks sold in surveyed LBMs were supplied by other LBMs, whereas broilers were equally sourced from other LBMs and farms, suggesting that more actors were involved in the trade of deshis and ducks than in the trade of broilers. Deshi and duck traders also travelled greater distances than broiler traders. This meant that deshi and duck movements could have been directly involved in the aforementioned long-distance HPAI H5N1 transmission events, and that these poultry types were likely to spend more time in traders’ hands than broiler chickens. They might thus greatly contribute to the amplification and maintenance of the viral circulation along the live poultry trading network [33]. Deshi and duck trade networks were less connected than other poultry type-specific networks. However, interactions between those networks – through the mixing of multiple poultry types in LBMs – meant that the connectedness of the overall network was very high. Almost all farming systems and production areas across the country were thus potentially epidemiologically connected through the network. Although ducks, and to a lesser extent deshis, represented a small proportion of the overall poultry trade in Bangladesh, the network characteristics may create conditions for viral strains circulating in an otherwise isolated deshi or duck population to spread to geographically distant poultry populations. Further research should aim to quantify the time spent by different poultry types within the trade network and, therefore, their potential to amplify the level and increase the diversity of viral circulation. Also, when multiple traders, and LBMs, are involved in the transport of poultry from the farm gate to the end-user, the actual production areas from which poultry originate should be identified. This would allow a better assessment of the way in which poultry type-specific trading practices may shape the overall network structure and contribute to its potential to spread AIVs.

## Conclusion

In conclusion, poultry trade practices varied according to the poultry type considered. It was the interaction between poultry type-specific networks that resulted in an overall live poultry trading network within which almost all poultry production areas across the country and LBMs identified during this survey were connected. While it appeared that control interventions targeted at a small number of key LBMs could be efficient and effective in controlling virus circulation, the feasibility of this strategy, taking into account the likelihood of behaviour change amongst all actors involved, would require further investigation.

## Additional file

Additional file 1: Table S1. Total number of poultry that interviewed traders reported trading in the week prior to the interviews. As the proportion of traders interviewed per surveyed LBM is unknown the actual number of poultry traded weekly at each LBM could not be estimated. (DOCX 12 kb)

## Abbreviations

AI: Avian influenza
AIV: Avian influenza virus
CCC: Chittagong corporation
DCC: Dhaka city corporation
GPS: Global positioning system
GSC: Giant strong component
GWC: Giant weak component
HPAI H5N1: Highly pathogenic avian influenza
IQR: Inter-quantil range
LBM: Live bird market
