# Supplementary material for: A large-scale study of a poultry trading network in Bangladesh: implications for control and surveillance of avian influenza viruses
Source: BMC Vet Res. 2018 Jan 12;14:12. doi: 10.1186/s12917-018-1331-5 (PMC5767022; doi:10.1186/s12917-018-1331-5)
Supplement: Additional file 1: Table S1. — Total number of poultry that interviewed traders reported trading in the week prior to the interviews. As the proportion of traders interviewed per surveyed LBM is unknown the actual number of poultry traded weekly at each LBM could not be estimated. (DOCX 12 kb) [file 12917_2018_1331_MOESM1_ESM.docx]

**ADDITIONAL FILE 1**

Table S1: Total number of poultry that interviewed traders reported trading in the week prior to the interviews.

As the proportion of traders interviewed per surveyed LBM is unknown the actual number of poultry traded weekly at each LBM could not be estimated.

|  | Broiler | Sonali | Deshi | Ducks | Others | All |
| --- | --- | --- | --- | --- | --- | --- |
| Number (%) of poultry sold by all interviewed traders | 1 020 256 (42.3%) | 795 939 (33%) | 461 402 (19.1%) | 12 806 (0.53%) | 123 265 (5.1%) | 2 413 668  (100%) |
